# TGF-β1 promotes scar fibroblasts proliferation and transdifferentiation via up-regulating MicroRNA-21

**DOI:** 10.1038/srep32231

**Published:** 2016-08-24

**Authors:** Ying Liu, Yue Li, Ning Li, Wen Teng, Min Wang, Yingbo Zhang, Zhibo Xiao

**Affiliations:** 1 Department of Plastic and Aesthetic Surgery, The Second Affiliated Hospital of Harbin Medical University, Harbin 150081, People’s Republic of China; 2 Department of General Surgery, The Second Affiliated Hospital of Harbin Medical University, Harbin 150081, People’s Republic of China

## Abstract

TGF-β1, upregulated in keloid tissue, promotes the proliferation, collagen formation and differentiation of dermal fibroblasts. miR-21 is one of microRNAs first found in human genome. The aim of our study is to explore the mechanisms of miR-21 in TGF-β1-induced scar fibroblasts proliferation and transdifferentiation. In the present study, first we found that TGF-β1 promoted scar fibroblasts proliferation and transdifferentiation via up-regulating miR-21 expression, which could be attenuated when miR-21 was inhibited. Overexpression of miR-21 had similar effect as TGF-β1 on proliferation and transdifferentiation. Additionally, TGF-β1 increased the expressions and activities of MMP2 and MMP9 in keloid fibroblasts, which was suppressed by miR-21 inhibition. Finally, the results demonstrated that PTEN/AKT signaling pathway played important role in TGF-β1-induced transdifferentiation. In conclusion, our study suggests that TGF-β1 promotes keloid fibroblasts proliferation and transdifferentiation via up-regulation of miR-21 and PTEN/AKT signalling pathway plays important role in this process, which provides a potential theoretical basis for clinical treatment of skin scars.

Scar, caused by various trauma, is the change of appearance and histopathology of normal skin. Keloid and hyperplastic scar are the common seen pathological scars in clinics and are also the difficult challenges in plastic surgery^1^,^2^. The main characters of pathological scar are fibroblast proliferation and excessive accumulation of extracellular matrix^3^. In recent years, although the pathological scar is brought to lots of valuable researches by the scientists in the world, its formation mechanisms are not quite clear. It is now generally accepted that the abnormal proliferation of fibroblast is one of the main causes of hyperplasia and persistence of pathological scar^4^. Therefore, the research on pathological scar formation mechanism is of great importance to explore the new treatments for pathological scar.

Transforming growth factor-β1 (TGF-β1), the important member of TGF-β family, can regulate the growth and differentiation of cells. Researches have shown that TGF-β1 was upregulated in keloid tissue, which promoted the proliferation, collagen formation and differentiation of dermal fibroblasts^5^,^6^,^7^. However, the detailed mechanisms of TGF-β1 in keloid formation are not very clear up to now, which need to be further studied. Recent studies have suggested that TGF-β1 could induce the expression of miR-21 in human umbilical vein endothelial cells and the mesangial cells^8^,^9^. miR-21 is upregulated in keloid tissue and could affect the proliferation and apoptosis of skin fibroblast via regulating the expression of its target gene PTEN^10^,^11^. Based on the above study background, we speculate that TGF-β1 may promote keloid formation though up-regulating miR-21 in human keloid fibroblasts.

In this study we have investigated the effect of TGF-β1 on the proliferation and differentiation of human keloid fibroblasts via regulating the expression of miR-21 and elucidated the related potential molecular mechanisms.

## Results

### miR-21 is upregulated by TGF-β1 in human keloid fibroblasts, promoting proliferation and transdifferentiation

The effect of TGF-β1 on keloid fibroblasts proliferation was detected by MTT and BrdU assays. After treatment for 12 h, 24 h, and 48 h, TGF-β1 promoted keloid fibroblasts (see Supplementary Fig. S1(A,B)) and primary keloid fibroblasts (see Supplementary Fig. S2(A)) proliferation in a time-dependent manner. The transdifferentiation of keloid fibroblasts from fibroblast-to-myofibroblast induced by TGF-β1 was investigated subsequently. The marker of myofibroblast α-SMA and signal molecule of fibroblast E-cadherin were detected. In keloid fibroblasts (see Supplementary Fig. S1(C,D)) and primary keloid fibroblasts (see Supplementary Fig. S2(B–D)) TGF-β1 results in a time-dependent increase in the expression of α-SMA, as assayed by western blot. While the expression of E-cadherin was decreased significantly after treatment with TGF-β1. Results from what have been presented above suggested that TGF-β1 promoted the proliferation and transdifferentiation of keloid fibroblasts.

**Figure 1:**
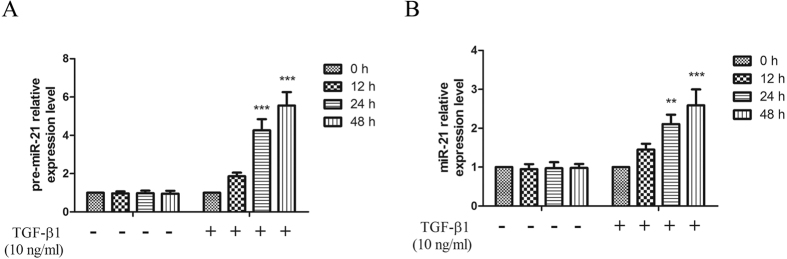
Up-regulating of miR-21 was induced by TGF-β1 in human keloid fibroblasts. The expressions of pre- (**A**) and mature miR-21 (**B**) in human keloid fibroblasts after treatment with TGF-β1 for different time were detected by real-time PCR. Each result represents at least three independent experiments. Data represents the mean ± SD (n = 3). **P < 0.01, ***P < 0.001, versus the control group.

To test whether miR-21 was involved in TGF-β1-induced proliferation and transdifferentiation of keloid fibroblasts, the expression of pre- and mature miR-21 was observed. TGF-β1 remarkably increased the expression levels of both pre- and mature miR-21 in a time-dependent manner in keloid fibroblasts (Fig. 1(A,B)) and primary keloid fibroblasts (see Supplementary Fig. S2(E,F)).

**Figure 2:**
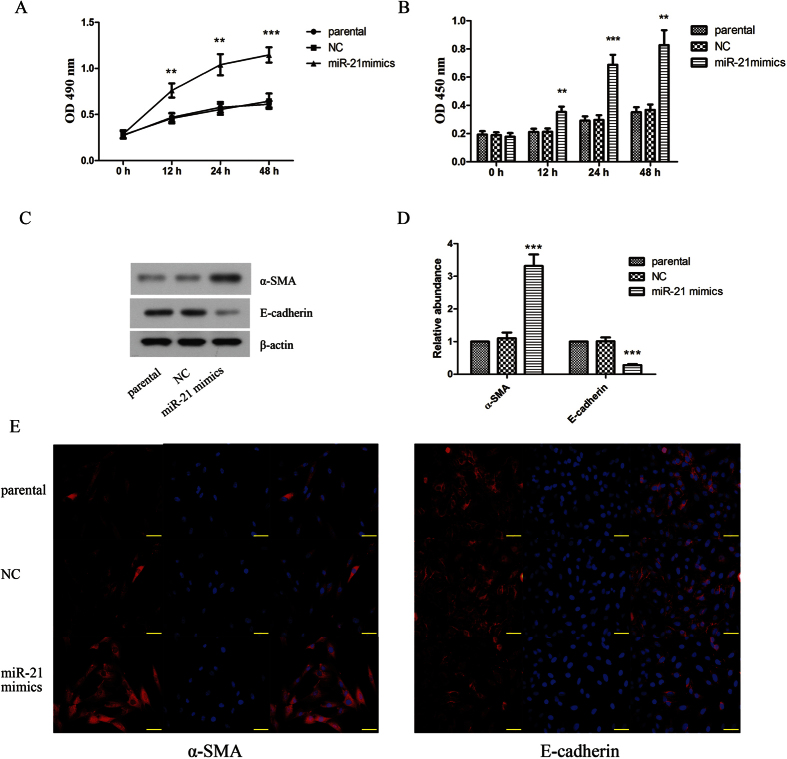
Overexpression of miR-21 promoted proliferation and transdifferentiation in human keloid fibroblasts. Cell proliferation was determined by MTT (**A**) and BrdU (**B**) assays. (**C,D**) α-SMA was up-regulated and E-cadherin was down-regulated by miR-21 overexpression assayed by western blot. β-actin was used as a loading control. (**E**) The protein expressions of α-SMA and E-cadherin were detected by immunofluorescence staining. Each result represents at least three independent experiments. Data represents the mean ± SD (n = 3). **P < 0.01, ***P < 0.001, versus the parental group.

**Figure 3:**
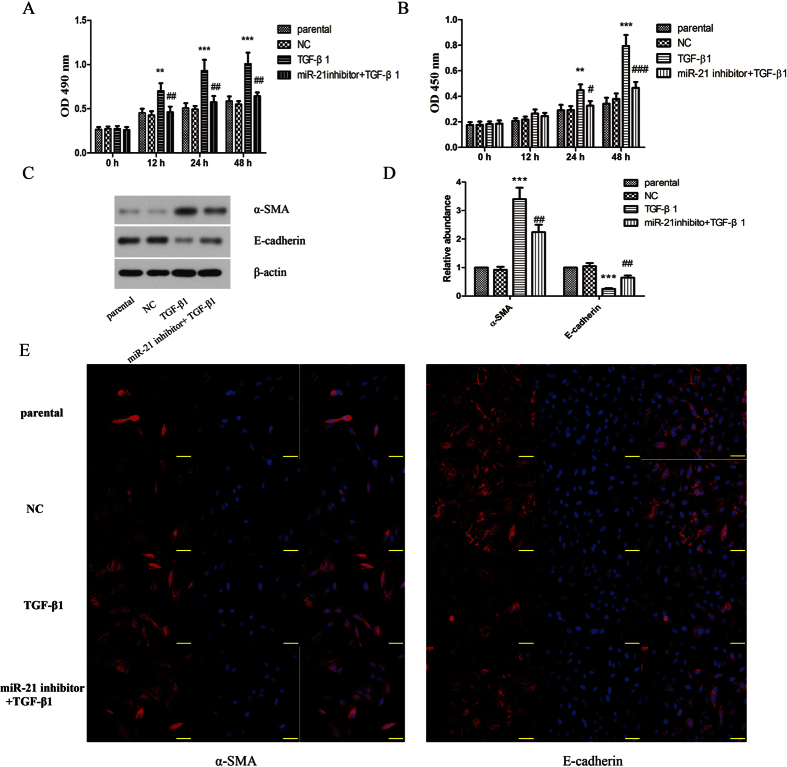
TGF-β1 induced-proliferation and transdifferentiation in human keloid fibroblasts was attenuated by miR-21 inhibition. Cell proliferation was determined by MTT (**A**) and BrdU (**B**) assays. (**C**) The protein expressions of α-SMA and E-cadherin were determined by western blot assay. (**D**) The protein quantification histogram was shown. β-actin was used as a loading control. (**E**) The protein expressions of α-SMA and E-cadherin were determined by immunofluorescence staining. Each result represents at least three independent experiments. Data represents the mean ± SD (n = 3). **P < 0.01, ***P < 0.001, versus the parental group. ^##^P < 0.01, ^###^P < 0.001, versus the TGF-β1 group.

Then we further elaborated the effect of miR-21 on proliferation and transdifferentiation of keloid fibroblasts. The result showed that overexpression of miR-21 significantly promoted keloid fibroblasts (Fig. 2(A,B)) and primary keloid fibroblasts (see Supplementary Fig. S3(A)) proliferation. Moreover, we also investigated the effect of miR-21 on transdifferentiation. As shown in Fig. 2(C–E), the results of western blot and immunofluorescence staining demonstrated that overexpression of miR-21 up-regulated the level of α-SMA and down-regulated the level of E-cadherin. In addition, inhibition of miR-21 suppressed TGF-β1-induced proliferation of keloid fibroblasts (Fig. 3(A,B)) and primary keloid fibroblasts (see Supplementary Fig. S3(B)). As shown in Fig. 3(C–E), the TGF-β1-induced up-regulation of α-SMA and down-regulation of E-cadherin could be restrained by inhibition of miR-21. From these above results, we could make a conclusion that miR-21 had effect similar to TGF-β1 on promoting proliferation and transdifferentiation of keloid fibroblasts.

### miR-21 is involved in TGF-β1-induced expressions and activities of MMPs

**Figure 4:**
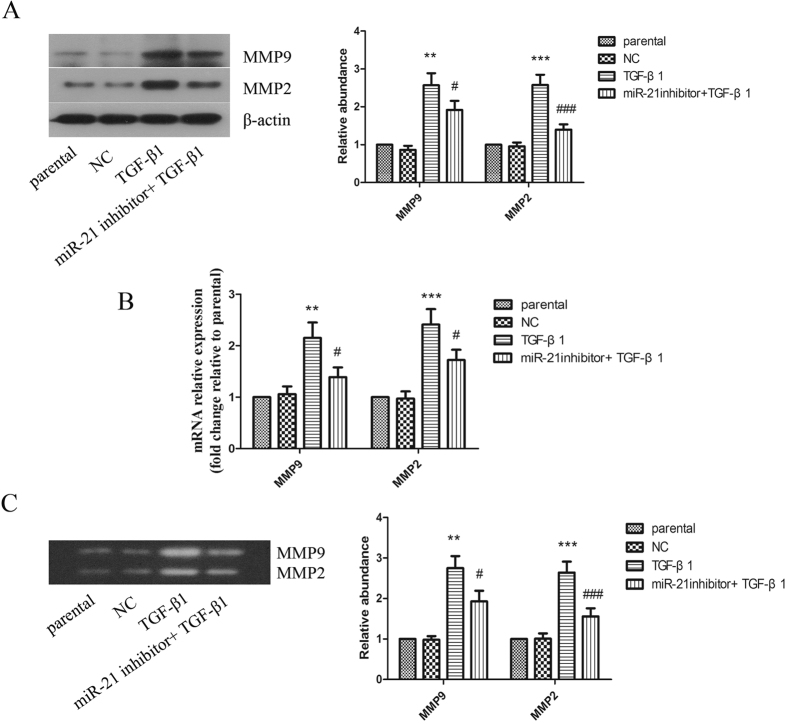
TGF-β1 induced-expressions and activities of MMP2 and MMP9 in human keloid fibroblasts was attenuated by miR-21 inhibition. (**A**) The protein expressions of MMP9 and MMP2 were determined by western blot. The protein quantification histogram was shown. β-actin was used as a loading control. (**B**) The mRNA expressions of MMP9 and MMP2 were determined by real-time PCR. (**C**) The activities of MMP9 and MMP2 were detected by gelatin zymography assay. Each result represents at least three independent experiments. Data represents the mean ± SD (n = 3). **P < 0.01, ***P < 0.001, versus the parental group. ^#^P < 0.05, ^###^P < 0.001, versus the TGF-β1 group.

Since MMPs play important role in scar formation, we assessed the effect of miR-21 on TGF-β1-induced expressions and activities of MMP9 and MMP2. As shown in Fig. 4(A) and Supplementary Fig. S4(A,B), the levels of MMP9 and MMP2 were up-regulated by TGF-β1, which could be down-regulated significantly when miR-21 was inhibited in keloid fibroblasts and primary keloid fibroblasts. Inhibition of miR-21 also suppressed TGF-β1-induced mRNA expressions of MMP9 and MMP2 remarkably (Fig. 4(B) and Supplementary Fig. S4(C)). Moreover, we found similar results in MMP9 and MMP2 activities. As assayed by gelatin zymography and showed in Fig. 4(C), TGF-β1 significantly promoted the activities of MMP9 and MMP2, which also could be restrained by miR-21 inhibition.

### AKT signaling pathway plays pivot roles in TGF-β1-induced transdifferentiation

**Figure 5:**
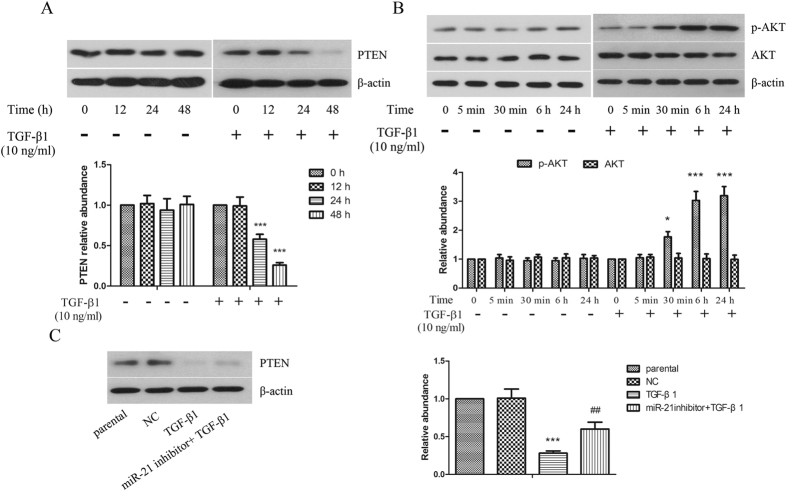
PTEN/AKT signaling pathway was regulated by TGF-β1 in human keloid fibroblasts, which could be weakened by miR-21 inhibition. The protein expressions of PTEN (**A**) and AKT (**B**) after treatment with TGF-β1 for different time were determined by western blot. β-actin was used as a loading control. (**C**) Inhibition of miR-21 attenuated the down-regulation of PTEN induced by TGF-β1. β-actin was used as a loading control. Each result represents at least three independent experiments. Data represents the mean ± SD (n = 3). *P < 0.05, ***P < 0.001, versus the parental group. ^##^P < 0.01, versus the TGF-β1 group.

**Figure 6:**
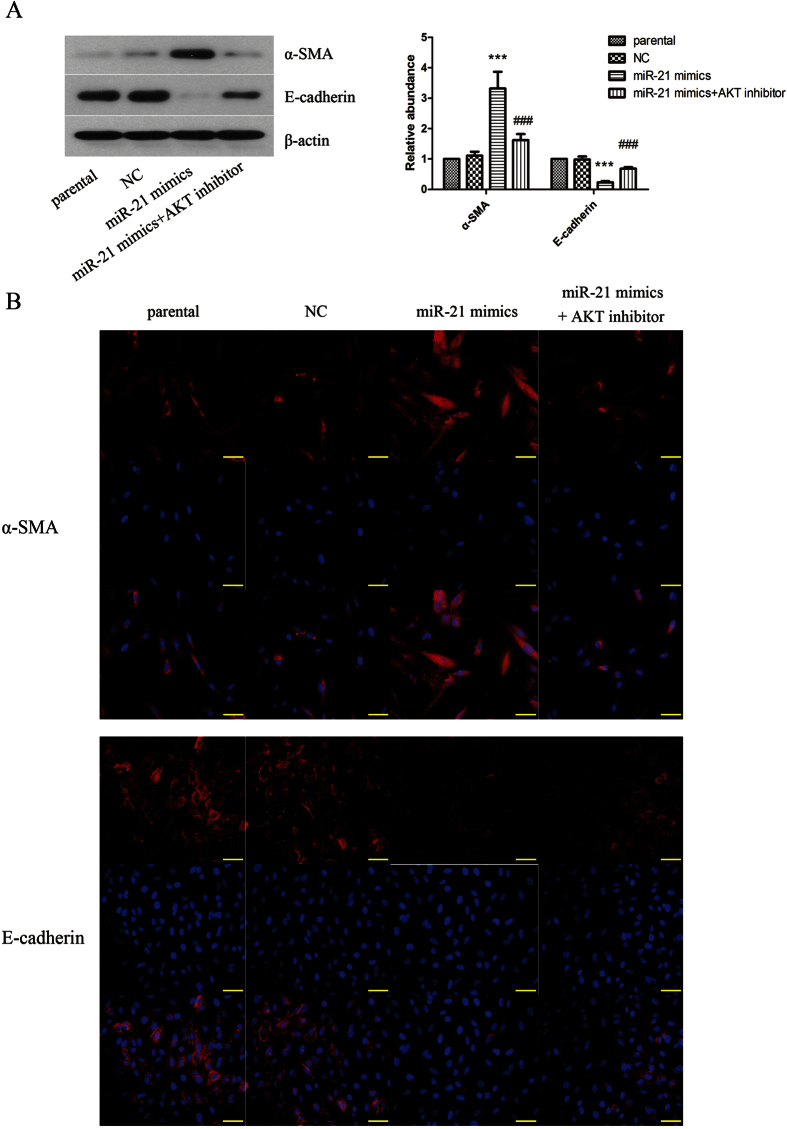
Inhibiting the expression of AKT could restrain the transdifferentiation induced by miR-21 overexpression in keloid fibroblasts. (**A**) The protein expressions of α-SMA and E-cadherin were determined by western blot assay. The protein quantification histogram was shown. β-actin was used as a loading control. (**B**) The protein expressions of α-SMA and E-cadherin were determined by immunofluorescence staining. Each result represents at least three independent experiments. Data represents the mean ± SD (n = 3). ***P < 0.001, versus the parental group. ^###^P < 0.001, versus the TGF-β1 group.

To further explore the mechanisms of miR-21 in TGF-β1-induced transdifferentiation, we focused on PTEN, which is the validated miR-21 target. The levels of PTEN in keloid fibroblasts (Fig. 5(A) and primary keloid fibroblasts (see Supplementary Fig. S5(A)) were down-regulated by treatment with TGF-β1 obviously. AKT is reported to be negatively regulated by PTEN, so we detected the expression of AKT subsequently. As shown in Fig. 5(B) and Supplementary Fig. S5(B), with the action time of TGF-β1 extending, the expression of phosphorylated AKT proteins was increased significantly in keloid fibroblasts and primary keloid fibroblasts. In addition, miR-21 inhibition attenuated the down-regulation of PTEN induced by TGF-β1 (Fig. 5(C) and Supplementary Fig. S5(C)). Moreover, we found that inhibiting the expression of AKT could restrain the up-regulation of α-SMA and down-regulation of E-cadherin induced by miR-21 overexpression in keloid fibroblasts, as evidenced by western blot and immunofluorescence staining assay (Fig. 6(A,B)).

## Discussion

Scar formation is a complex biological process involving multiple cell signaling pathways and cytokines. TGF-β1 is recognized as an important growth factor, which promotes the progress of tissue fibrosis. Compared with fibroblasts of normal skin, the expression of TGF-β1 in hypertrophic scar tissues and fibroblasts is detected at high level^12^. Shah *et al.* found that neutralisation of TGF-β1 could reduce scar formation in wounded rats^13^, which provided direct evidence that TGF-β1 could cause scar formation. Subsequently, Loiselle *et al.* suggested that modulation of the TGF-β signaling pathway can prevent scarring during flexor tendon repair^14^. miR-21 is one of microRNAs first found in human genome and is also the only one upregulated in all human malignant tumors^15^,^16^. So a large number of researches have focused on the physiological and pathological functions of miR-21^17^,^18^,^19^,^20^. Recently, the role of miR-21 in fibrotic process has attracted more and more scholars’ attention. Previous study found that the expression of miR-21 could be induced by TGF-β1^21^, which hints that miR-21 may play significant role in TGF-β1-induced scar formation. In our present study, we aimed at exploring the effect of miR-21 on TGF-β1-induced proliferation and transdifferentiation in scar fibroblasts.

Fibroblasts are major participants in wound repair, which take part in granulation tissue’s formation, collagen synthesis and interact with extracellular matrix, thereby promoting scar hyperplasia. Early in wound healing, fibroblasts synthesis and secrete a certain amount of collagen to accelerate wound healing. In the development of hypertrophic scar, massive proliferation of fibroblasts promotes collagen secretion and leads to a large number collagen deposition^22^. Meanwhile, a huge release of growth factor from fibroblasts can promote scar hyperplasia. Thus it can be seen that the uncontrolled proliferation of fibroblasts is the foundation to form scar. In our present study, after stimulation with TGF-β1 the proliferation of keloid fibroblasts was promoted significantly via up-regulation of miR-21. This is the evidence that miR-21 is involved in TGF-β1-induced scar formation.

Cells may have the morphological, structure and functional changes under special physiological and pathological condition, which is called transdifferentiation. The transdifferentiation of fibroblasts into myofibroblasts is one of the important mechanisms of scar formation. There are lots of myofibroblasts in hyperplastic scar tissues and α-SMA is the marker protein of myofibroblasts^23^. Researches have shown that myofibroblasts could increase the deposition of extracellular matrix and cause tissue shrink, which has close relation with the occurrence and development of organ fibrosis. Thus, the number and function of myofibroblasts largely determines the speed and extent of scar formation. Inhibiting the transdifferentiation of fibroblasts to myofibroblasts is one of the top tactics to stop the development of scar tissue. In this study, our results indicated that miR-21 functioned like TGF-β1, promoting the transdifferentiation of fibroblasts to myofibroblasts. Inhibition of miR-21 attenuated TGF-β1-induced transdifferentiation in keloid fibroblasts. All those results suggest that miR-21 plays important role in TGF-β1-induced keloid fibroblasts transdifferentiation.

The histological features of keloids are a large number of fibroblasts proliferation, excessive accumulation of extracellular matrix (ECM), and irregularly arranged collagen fibers. Matrix metalloproteinases (MMPs) are a family of proteolytic enzymes involved in the degradation of the ECM. MMP-2 and MMP-9, mainly distributed in scar tissue, are the key enzymes of collagen and ECM degradation. Collagen IV is the common substrate of them. In the development of scar, there is an imbalance between ECM synthesis and degradation. In addition, some MMPs inhibitors are validated targets of miR-21^24^,^25^, so miR-21 may pomote the expressions and activities of MMPs by suppression MMPs inhibitors, which has been proved in mumerous studies. Zhou *et al.* found that STAT3 inhibitor reduced miR-21 expression and also resulted in decreased expression of MMP 2 in human oral squamous cell carcinoma^26^. Research also demonstrated that the expression and secretion of MMP 9 was promoted by miR-21 in macrophages^27^. In our study, we found that the expressions and activites of MMP9 and MMP2 were up-regulated by TGF-β1, which could be down-regulated significantly when miR-21 was inhibited.

Phosphatase and tensin homolog (PTEN) is deleted or mutated in several types of human cancer and is considered to be a tumor suppressor gene^28^,^29^. Recent evidence has also confirmed that the protein expression of PTEN could be suppressed by microRNAs and PTEN is confirmed to be target for miR-21^30^,^31^. Research also found that PTEN could reduce the phosphorylation of AKT protein^32^, which was involved in hypertrophic scar formation^33^. Moreover, the expression of PTEN could be effectively inhibited by TGF-β^34^. Based on the above results, we speculate that miR-21 may play an important role in scar fibroblasts transdifferentiation through PTEN/AKT pathway. Our results indicated that PTEN was down-regulated by TGF-β1 in keloid fibroblasts, which could be restrained by miR-21 inhibition. AKT protein is an important member of survival pathway, but is not fully functional in autonomously growing cell lines. However, in our study the keloid fibroblasts were treated with TGF-β1 to simulate the pathologic condition of fibrosis. According to our result, the expression of phosphorylated AKT proteins was increased significantly after treatment with TGF-β1. In addition, inhibition of AKT signaling pathway could suppress transdifferentiation in keloid fibroblasts induced by miR-21 overexpression. The above results are evidence of the critical role of PTEN/AKT signaling pathway in TGF-β1-induced transdifferentiation via up-regulation of miR-21.

So far, there are mainly four different delivery systems of miRNA-based therapeutics, such as through viral vectors, lipid-based systems, nanocarriers and LNA-based compounds delivery^35^. The possible side effects of blocking miR-21 have been rarely reported. Security is an urgent issue for the miRNA-based therapeutic delivery and a considerable amount of studies are needed in the future.

In summary, our study demonstrates that TGF-β1 promotes keloid fibroblasts proliferation and transdifferentiation via up-regulation of miR-21 and PTEN/AKT signaling pathway plays pivotal role in this process. Our findings provide a novel theoretical basis for prevention and treatment of skin scars.

## Methods

### Cell line and reagent

Human keloid fibroblasts line was purchased from Aiyan Biotech Co., Ltd (Shanghai, China), which was obtained from surgically resected keloid tissue. The cells were cultured in DMEM (Gibco, USA) and supplemented with 10% fetal bovine serum (Hyclone, USA), containing 100 μg/ml streptomycin and 100 U/ml penicillin (Hyclone, USA), at 37 °C, under a 5.0% CO2 atmosphere. GSK690693, a pan-AKT inhibitor, was purchased from Selleck China (Shanghai, China).

### Primary keloid fibroblasts culture

Keloid tissues were washed with phosphate-buffered saline (PBS), cut into small pieces, adhered and cultured on the bottom of tissue culture flasks. After 3–5 d of culture, the fibroblasts grew out of tissues. Primary keloid fibroblasts were collected and then cultured in DMEM (Gibco, USA) supplemented with 10% fetal bovine serum (Hyclone, USA), containing 100 μg/ml streptomycin and 100 U/ml penicillin, at 37 °C, under a 5.0% CO2 atmosphere. Primary keloid fibroblasts used in this study were retained in the fourth to seventh passage.

### Transient transfection

The miR-21 mimics, mimics negative control (mimics control), miR-21 inhibitor, inhibitor negative control (inhibitor control) were purchased from Genepharma Inc (Shanghai, China). Cells were transfected with oligonucleotides by Lipofectamine 2000 (Invitrogen, USA) at a final concentration of 100 nM following the manufacturer’s instructions. At the indicated time for various assays cells were collected.

### Cell proliferation assay

MTT and BrdU assays were performed to measure cell proliferation. Briefly, cells cultured in DMEM (10% FBS) were seeded into 96-well plates and treated with 10 ng/ml TGF-β for 0 h, 12 h, 24 h, and 48 h or transfected with miRNA-21 mimics or inhibitor and treated with 10 ng/ml TGF-β for 0 h, 12 h, 24 h, and 48 h. For MTT assay, 20 μl MTT was added into each well. After incubation for 4 h and removing the media, 200 μl DMSO was added into each well to dissolve the formazan crystalline product. The results were measured at 490 nm using a microplate reader (Bio Tek, USA). For BrdU assay, 10 μM BrdU labelling reagent (Maibio, China) was added into each well. After 2 h, the incorporated BrdU was detected using peroxidase-labeled monoclonal anti-BrdU-POD. The results were visualised with the soluble substrate solution and measured at 450 nm.

### RNA extraction and real-time PCR

TRIzol Reagent (Invitrogen, USA) was used to extract total RNA from the cells. cDNA was synthesized from five hundred ng of total RNA using a reverse transcriptase (TaKaRa, Japan). miRNAs were reverse transcribed to cDNA using miScript Reverse Transcription Kit (Qiagen, Germany). RNA expression was measured by real-time PCR in ABI Prism 7500 Sequence Detection System (Applied Biosystems, USA) using the SYBR-Green method (Takara, Japan) according to the manufacturer’s instructions. The qPCR for pre-miR-21, miR-21 and endogenous control U6 was performed using Hairpin-it miRNAs qPCR quantitation kit and U6 snRNA real-time PCR normalization kit (GenePharma, China). The primer sequences were listed in Table 1. β-actin and U6 were used as internal reference genes. Relative quantification of gene was calculated by the comparative threshold cycle method.

### Antibodies and Western blot

A mouse anti-α-SMA (1:400) antibody was purchased from Boster (Wuhan, China). Rabbit anti- E-cadherin (1:400), anti-MMP2 (1:400), anti-MMP9 (1:400) were obtained from Boster (Wuhan, China). Rabbit anti-PTEN (1:500) were obtained from Bioss(Woburn, USA). Rabbit anti- pAKT (1:200), anti-AKT (1:200), anti-β-actin(1:1000) were obtained from Santa Cruz (Dallas, USA). Briefly, the cells were lysed on ice in a RIPA lysis buffer (Beyotime, China) and then denatured. The amount of protein was quantified by the BCA protein assay kit (Beyotime, China). SDS-PAGE was used to separate equal quantities protein samples and then proteins were transferred to polyvinylidene fluoride membranes. After blocking with 5% dried skimmed milk in PBS for 1 h, the membranes were incubated with various specific primary antibodies respectively, at 4 °C overnight. Then they were incubated with secondary antibodies. The amount of bound antibody was detected by ECL detection reagent(Beyotime, China). The relative optical densities of the bands were measured to compared the expression levels of the target proteins among different groups. β-actin was served as a loading control.

### Immunofluorescence staining

Cells received different treatments were plated to slides at a density of 5 × 10^5^ in 6-well plates for 48 h. The cells were fixed with 4% paraformaldehyde for 15 min and permeabilized with 0.1% Triton X-100 for 30 min at room temperature. After blocking with 10% normal goat serum for 15 min, the cells were incubated with primary antibodies overnight at 4 °C respectively, and then incubated with Cy3-conjugated secondary antibodies for 1 h at room temperature. DAPI was used to stain nucleus. Under 400x magnification, images were randomly acquired by fluorescence microscope (Olympus, Japan) at 5 different microscopic fields.

### Gelatin Zymography

The activities of MMP2 and MMP9 were determined by gelatin zymography assay. The 30 μg total proteins of different groups were separated in a 10% SDS polyacrylamide gel containing 0.1% gelatin in an ice bath. After electrophoresis, the gels were washed in wash buffer (2.5% Triton X-100, 50 mM Tris-HCl, 5 mM CaCl2, 1 μM ZnCl2, pH7.6) for 40 min twice; incubated for 18 h at 37 °C in incubation buffer (50 mM Tris-HCl, 5 mM CaCl2, 1 μM ZnCl2, 0.02% Brij, 0.2 M NaCl). Then the gel was incubated with 0.05% (w/v) Coomassie brilliant blue R-250 and then destained with destaining solution (50% methanol, 10% acetic acid). The gel was photographed and protease activity was detected by scanning densitometry.

### Statistical analysis

All experimental values are expressed as the mean ± standard deviation (SD). The Student’s t-test was used to evaluate the means of two groups. Values between groups were analyzed by one-way ANOVA followed by Bonferroni’s Multiple Comparison Test using GraphPad Prism software. P < 0.05 was considered statistical significant.

## Additional Information

**How to cite this article**: Liu, Y. *et al.* TGF-β1 promotes scar fibroblasts proliferation and transdifferentiation via up-regulating MicroRNA-21. *Sci. Rep.* **6**, 32231; doi: 10.1038/srep32231 (2016).

## Supplementary Material

Supplementary Information

## Figures and Tables

**Table 1:** Oligonucleotide primer sets for real-time PCR.

Name	Sequence(5′¬3′)	Length
MMP 9 F	GCTACGTGACCTATGACATCCT	22
MMP 9 R	TCCTCCAGAACAGAATACCAGT	22
MMP 2 F	TGCTGAAGGACACACTAAAG	20
MMP 2 R	GTAGCCAATGATCCTGTATGT	21
miR-21 F	GGCAGCCTAGCTTATCAGACT	21
miR-21 R	GTGCAGGGTCCGAGGTATTC	20
pre-miR-21 F	GTGACCGCAACACCAGTCGATG	22
pre-miR-21 R	CTGGTGCAGGGTCCGAGGTATT	22
β-actin F	CTTAGTTGCGTTACACCCTTTCTTG	25
β-actin R	CTGTCACCTTCACCGTTCCAGTTT	24
U6 F	CTCGCTTCGGCAGCACA	17
U6 R	AACGCTTCACGAATTTGCGT	20
