# Supplementary material for: TGF-β1 promotes scar fibroblasts proliferation and transdifferentiation via up-regulating MicroRNA-21
Source: Sci Rep. 2016 Aug 24;6:32231. doi: 10.1038/srep32231 (PMC4995376; doi:10.1038/srep32231)
Supplement: Supplementary Information [file srep32231-s1.pdf]

TGF-β1 promotes scar fibroblasts proliferation and transdifferentiation via up-regulating MicroRNA-21

Ying Liu[1, #], Yue Li[2, #], Ning Li[1, #] Wen Teng[1], Min Wang[1], Yingbo Zhang[1], Zhibo Xiao[1, *]

[1] Department of Plastic and Aesthetic Surgery, The Second Affiliated Hospital of Harbin Medical University, Harbin 150081, People's Republic of China

[2] Department of General Surgery, The Second Affiliated Hospital of Harbin Medical University, Harbin 150081, People's Republic of China

* Corresponding author: Dr. Zhibo Xiao,

e-mail address: xiaozhibodoctor@126.com

Address: Department of Plastic and Aesthetic Surgery, The Second Affiliated Hospital of Harbin Medical University, 148 Baojian Road, Harbin 150081, People's Republic of China

Tel: +86-451-86297103

[#] Ying Liu,Yue Li and Ning Li contributed equally to this study.

Supplementary Figure S1

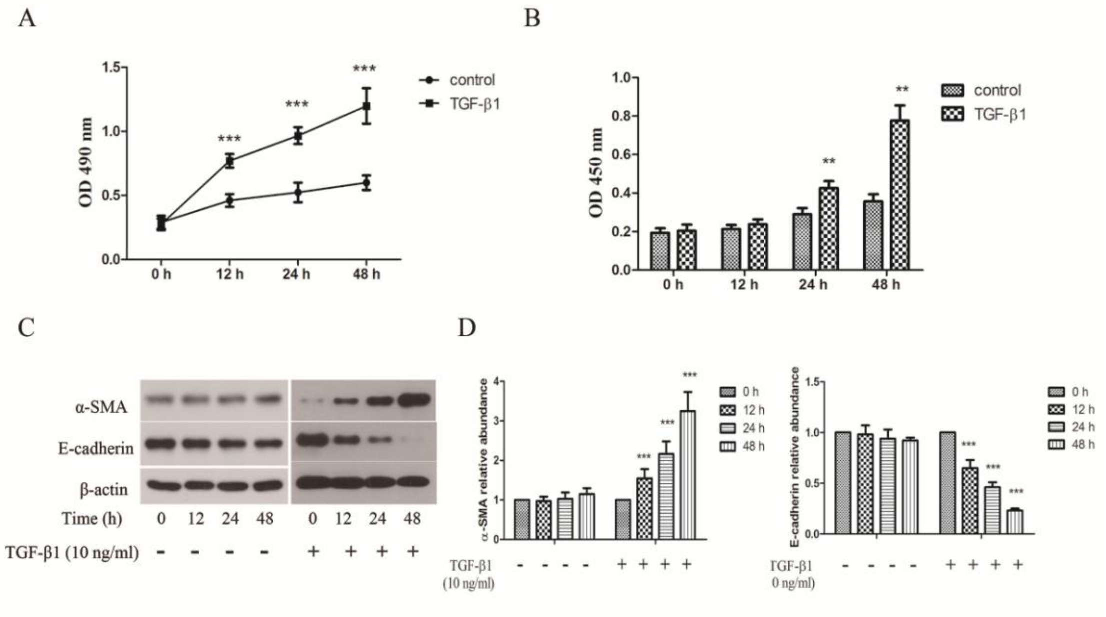

Supplementary Figure S2

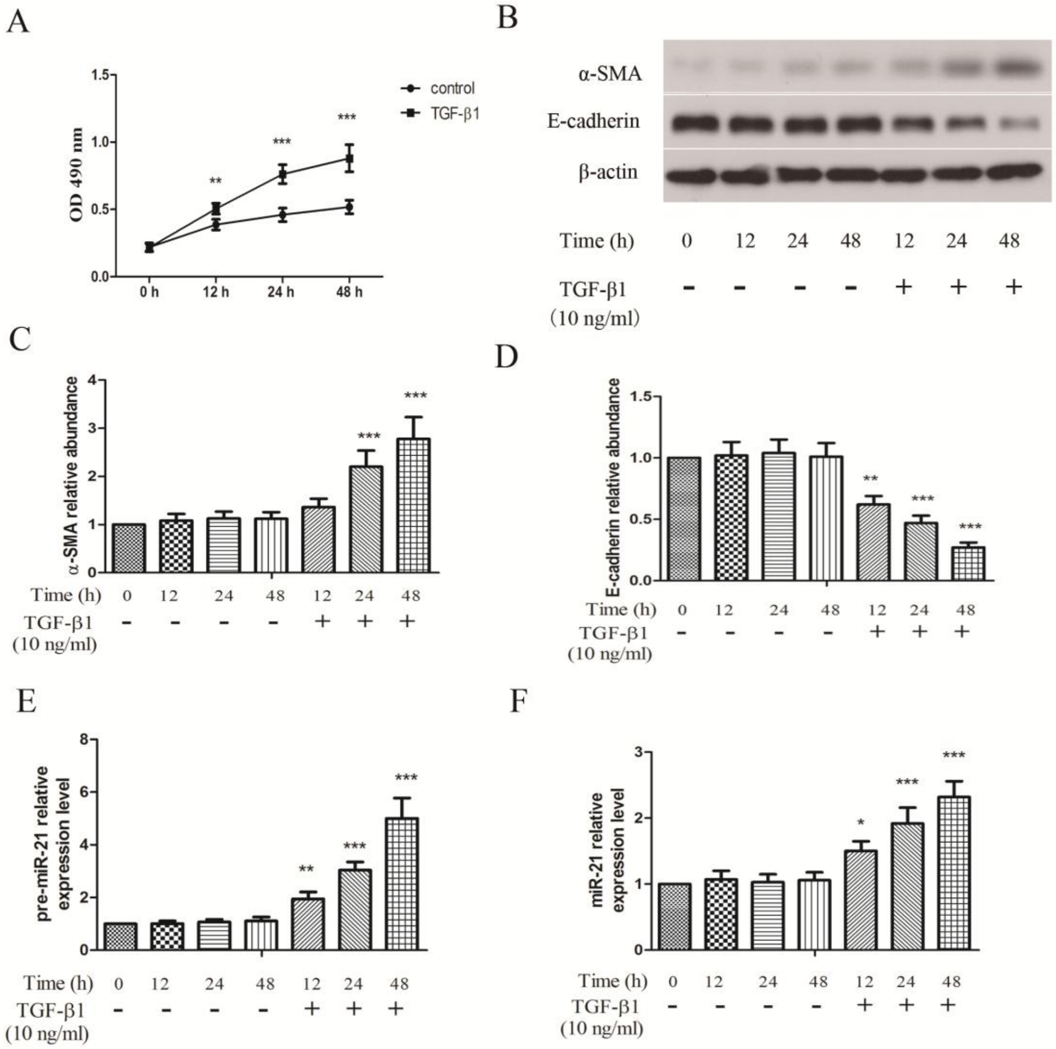

Supplementary Figure S3

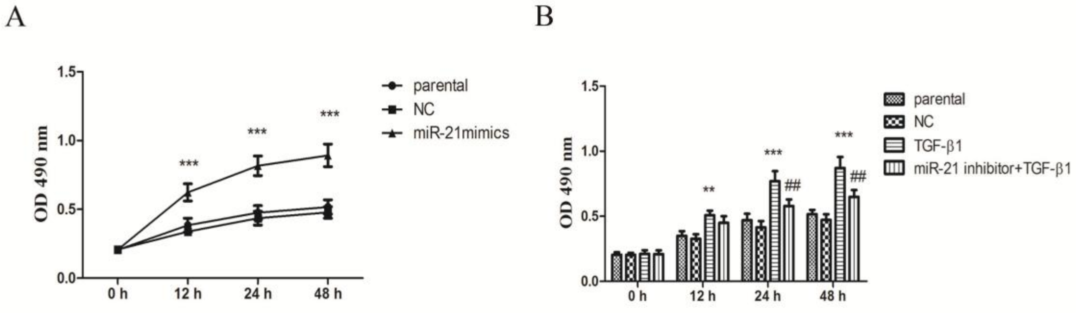

Supplementary Figure S4

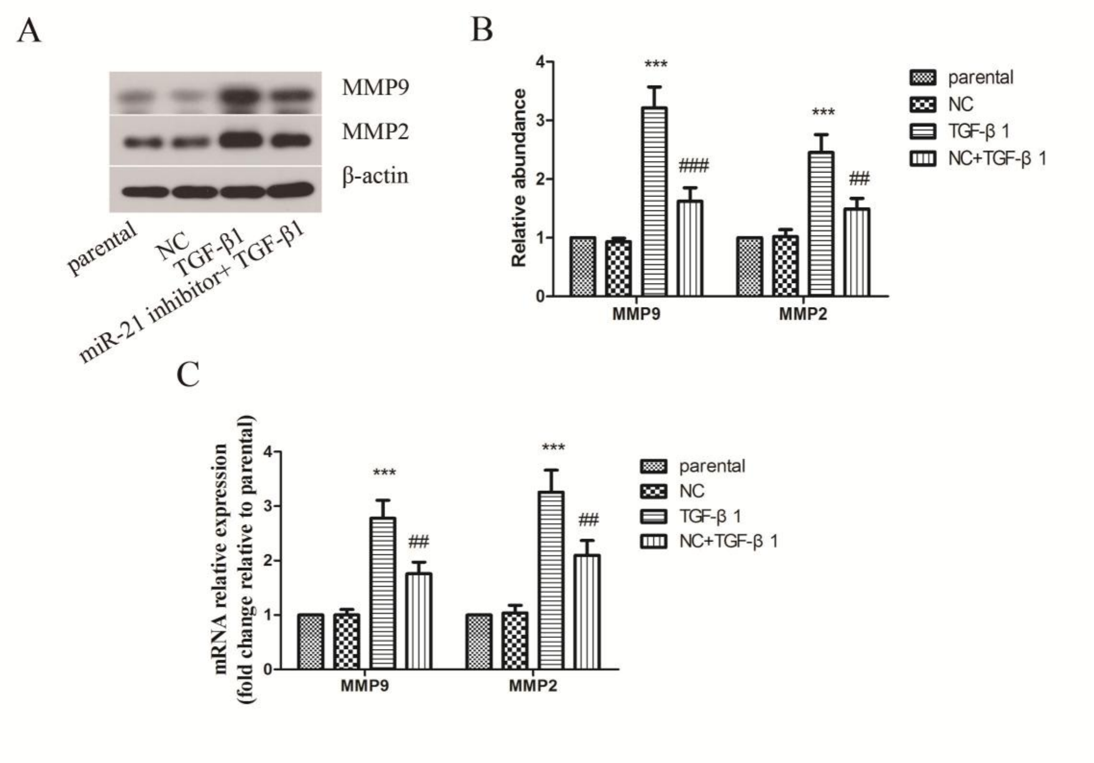

Supplementary Figure S5

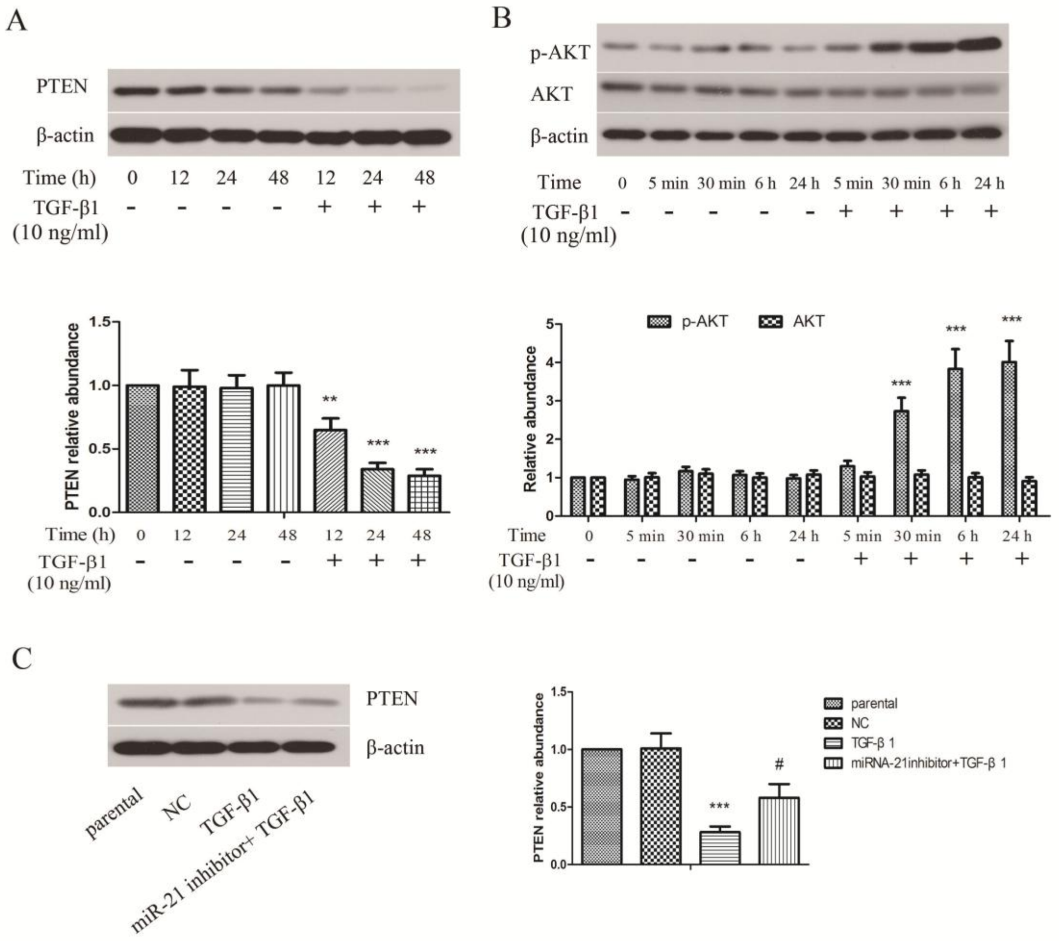

Supplementary Figure S1. TGF-β1 induced proliferation and transdifferentiation in human keloid fibroblasts. Cell proliferation was detected by MTT (A) and BrdU (B) assays at different time points. (C) The α-SMA and E-cadherin protein expressions were determined by western blot assay. (D) The protein quantification histogram was shown. β-actin was used as a loading control. Each result represents at least three independent experiments. Data represents the mean ± SD (n = 3). **P < 0.01, ***P < 0.001, versus the control group.

Supplementary Figure S2. TGF-β1 induced proliferation and transdifferentiation in primary human keloid fibroblasts via up-regulating miR-21. (A) Cell proliferation

was detected by MTT assay at different time points. (B) The α-SMA and E-cadherin protein expressions were determined by western blot assay. (C-D) The protein quantification histogram was shown. β-actin was used as a loading control. The expressions of pre- (E) and mature miR-21 (F) in primary human keloid fibroblasts after treatment with TGF-β1 for different time were detected by real-time PCR. Each result represents at least three independent experiments. Data represents the mean ± SD (n = 3). $^{*}P < 0.05$, $^{**}P < 0.01$, $^{***}P < 0.001$, versus the control group.

Supplementary Figure S3. miR-21 promoted proliferation in primary human keloid fibroblasts. (A) Cell proliferation effected by miR-21 mimics was detected by MTT assay at different time points. (B) miR-21 inhibitor restrained TGF-β1 induced-proliferation in primary human keloid fibroblasts. Each result represents at least three independent experiments. Data represents the mean ± SD (n = 3). $^{**}P < 0.01$, $^{***}P < 0.001$, versus the parental group. $^{\#\#}P < 0.01$, versus the TGF-β1 group.

Supplementary Figure S4. TGF-β1 induced-expressions of MMP2 and MMP9 in primary keloid fibroblasts was attenuated by miR-21 inhibition. (A) The protein expressions of MMP9 and MMP2 were determined by western blot. (B) The protein quantification histogram was shown. β-actin was used as a loading control. (C) The mRNA expressions of MMP9 and MMP2 were determined by real-time PCR. Each result represents at least three independent experiments. Data represents the mean ± SD (n = 3). $^{***}P < 0.001$, versus the parental group. $^{\#\#}P < 0.01$, $^{\#\#\#}P < 0.001$, versus the TGF-β1 group.

Supplementary Figure S5. PTEN/AKT signaling pathway was regulated by TGF-β1

in primary keloid fibroblasts, which could be weakened by miR-21 inhibition. The protein expressions of PTEN (A) and AKT (B) after treatment with TGF-β1 for different time were determined by western blot. β-actin was used as a loading control. (C) Inhibition of miR-21 attenuated the down-regulation of PTEN induced by TGF-β1. β-actin was used as a loading control. Each result represents at least three independent experiments. Data represents the mean ± SD (n = 3). $^{**}P < 0.01$, $^{***}P < 0.001$, versus the parental group. $^{\#}P < 0.05$, versus the TGF-β1 group.
